# Ocular Manifestations after Receiving COVID-19 Vaccine: A Systematic Review

**DOI:** 10.3390/vaccines9121404

**Published:** 2021-11-27

**Authors:** Yu-Kuei Lee, Yi-Hsun Huang

**Affiliations:** Department of Ophthalmology, National Cheng Kung University Hospital, College of Medicine, National Cheng Kung University, Tainan 704, Taiwan; vincent11909@hotmail.com

**Keywords:** adverse events, coronavirus disease 2019 (COVID-19), ophthalmology, vaccines

## Abstract

The coronavirus disease 2019 (COVID-19) pandemic has had profound and lasting consequences since 2019. Although vaccines against COVID-19 have been developed and approved under emergency use authorization, various adverse events have also been reported after COVID-19 vaccination. This review was undertaken to help clinicians recognize the possible manifestations and systemic pathogenesis, especially those related to the eye, after receiving COVID-19 vaccination. A systemic search was performed on 22 August 2021 through Embase, Medline, and Cochrane Library for publications on ocular manifestations after COVID-19 vaccination. Two case-control studies/retrospective cohort studies, one cross-sectional study, three case series, sixteen case reports, two images, and seven letters were included. Ocular manifestations after receiving COVID-19 vaccines may appear on the eyelid, cornea and ocular surface, retina, uvea, nerve, and vessel. The ocular manifestations occurred up to forty-two days after vaccination, and vaccine-induced immunologic responses may be responsible. Although the incidence rate of ocular symptoms is considerably lower in the vaccinated subjects than in COVID-19 patients, physicians should be aware of the possible associations between COVID-19 vaccines and ocular symptoms for the early diagnosis and treatment of vision problems or life-threatening complications.

## 1. Introduction

The coronavirus disease 2019 (COVID-19), caused by severe acute respiratory syndrome coronavirus 2 (SARS-CoV-2), resulted in an unprecedented pandemic worldwide. The European Medicines Agency (EMA) and the US Food and Drug Administration have approved emergency use authorization for several COVID-19 vaccines before the completion of their clinical trials. Currently, EMA has approved four vaccines that are protective against SARS-CoV-2 [1,2]. BNT162b2 (Pfizer/BioNTech, Mainz, Germany) [3] and mRNA-1273 (Moderna, Cambridge, MA, USA) [4] belong to the category of lipid nanoparticle (LNP)-formulated mRNA COVID-19 vaccines, while ChAdO×1 (University of Oxford/AstraZeneca, Oxford, UK) [5] and Ad26.COV2.S (Johnson & Johnson/Janssen, New Brunswick, NJ, USA) [6] belong to the category of adenovirus vector COVID-19 vaccines. Several adverse events, including ocular manifestations, have been reported after vaccination. In this study, we aimed to review all reported ocular side effects of the available COVID-19 vaccines.

## 2. Materials and Methods

This review was conducted in accordance with the Preferred Reporting Items for a Systematic Review and Meta-Analyses (PRISMA) guidelines [7].

### 2.1. Search Strategy

To comprehensively identify the ocular manifestations of COVID-19 vaccination, we systematically searched three databases, including Embase, Medline (Ovid), and Cochrane Central for studies on related topics.

We used the following keywords for searching the databases: ((“2019-nCoV” OR “nCoV-19” OR “nCoV-2019” OR “SARS-CoV-2” OR “SARS-CoV2” OR “SARSCoV2” OR “SARS2” OR “COVID” OR “coronavirus” OR “coronavirus disease 2019” OR “severe acute respiratory syndrome”) vaccine) AND (“eye” OR “ocular” OR “oculopathy” OR “oculomotor” OR “intraocular” OR “extraocular” OR “ophthalmic” OR “vision” OR “orbit” OR “cornea” OR “conjunctiva” OR “retina” OR “sclera” OR “vitreous” OR “diplopia” OR “metamorphopsia” OR “visual” OR “optic”)). All terms were used as words in titles, abstracts, and keywords.

We also searched the references of the included studies for more relevant articles. The literature research was conducted on 22 August 2021. The inclusion criteria were original articles on ocular manifestations of patients who received COVID-19 vaccine. Case reports, case series, letters, and editorials were also included. Studies without any information on ocular manifestations of patients who received COVID-19 vaccine were excluded. Included studies were categorized in this study and divided based on the involved part of the eye.

### 2.2. Study Selection

We performed study selection using EndNote ×9. Two review authors (Y.K.L. and Y.H.H.) independently screened all titles and abstracts based on the inclusion criteria and subsequently retrieved all relevant full-text articles for suitability assessment. Any discrepancies were resolved via discussion until consensus was reached.

**Figure 1:**
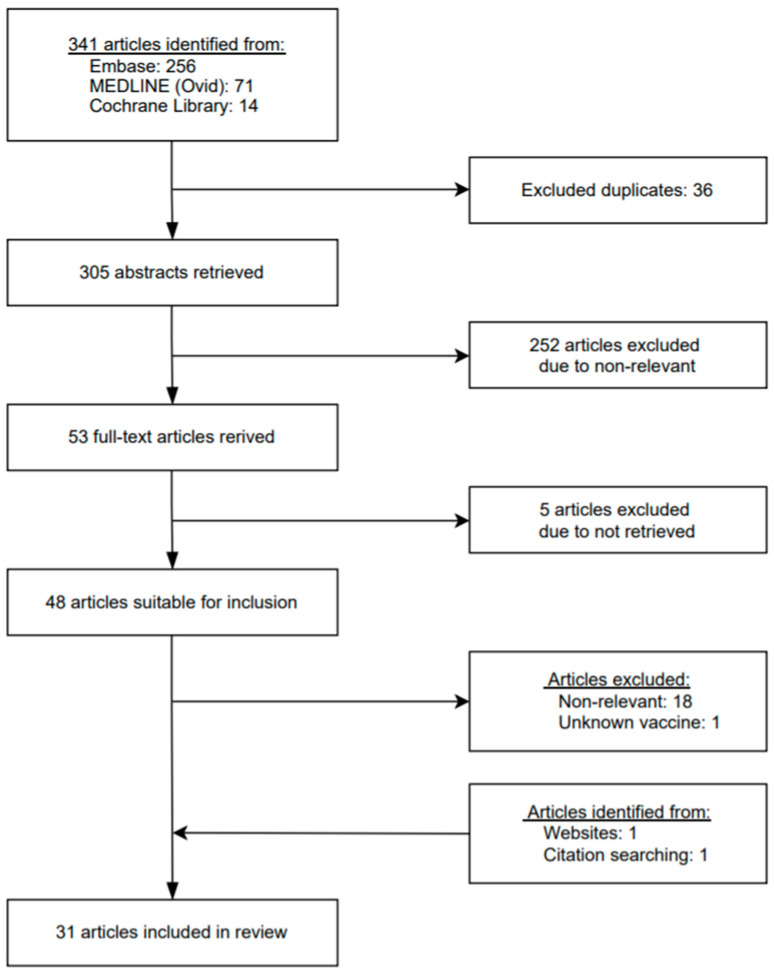
PRISMA flow diagram of the articles analyzed.

A flowchart depicting the process of retrieving articles is shown in Figure 1. A total of 341 studies were initially found after searching the databases according to the search strategy. The Embase, Medline and Cochrane Library searches yielded 256, 71 and 14 results respectively, of which 36 studies were excluded because of duplicate articles. We screened a total of 305 titles and abstracts for eligibility. Among these, 252 studies were excluded due to irrelevance and 53 studies were selected for full-text evaluation. We excluded 5 of these studies due to reports not being retrieved, leaving 48 articles suitable for inclusion. After full-text evaluation, 18 studies were excluded due to no relevant and 1 study was excluded due to unknown vaccine. On the other hand, 1 study was identified from websites, while another article was identified through a citation search. Finally, 31 reports matched the inclusion criteria of our study.

### 2.3. Data Extraction and Analysis

For each analyzed report, different ocular adverse events were recorded. An ocular adverse event was defined as any adverse event related to the eye, adnexa, or vision. In each report, the patient’s age, sex, history, name of vaccine received, date of first/second dose, duration of ocular adverse events that occurred after the vaccination, and fever were recorded. Quality rating of the studies was ranked according to Quality Rating Scheme for Studies and Other Evidence [8] and Oxford Centre for Evidence-based Medicine for ratings of individual studies [9]. Characteristics of ocular manifestations after receiving COVID-19 vaccine and their related reports are presented in Table 1.

## 3. Results

In total, 31 studies were included in our systemic review and classified into eyelid, cornea and ocular surface, retina, uvea, nerve, vascular thrombosis, and others according to different parts of eye and adjacent tissues.

### 3.1. Eyelid 

In our literature review, there were six patients with eyelid manifestations. The mean age of these patients was 57.5 (range 44–67) years old. The mean duration between COVID-19 vaccination and onset of ocular symptoms was 10.0 (range 1–21) days. Patient characteristics were reviewed in three articles.

- Eyelid swelling

In a study investigating symptoms after administration of three types of COVID-19 vaccines (Pfizer, AstraZeneca, and Sinopharm), Al Khames Aga et al. reported that two out of 1736 participants had eyelid swelling and severe allergic reaction on the day of vaccination with BNT162b2 COVID-19 vaccine [10].

- Eyelid purpuric lesions

In a case series study, Mazzatenta et al. reported that a 44-year-old female and a 63-year-old male had purpuric lesions on the upper eyelids occurring 21–25 days after vaccination with the second dose of BNT162b2 COVID-19 vaccine. The other case was a 67-year-old female who developed ecchymosis on the upper eyelids 10 days after the first dose of BNT162b2 COVID-19 vaccine. All three patients’ purpuric eyelid lesions were asymptomatic and resolved spontaneously after 10–15 days [11].

- Herpes Zoster Ophthalmicus (HZO)

Furer et al. conducted a case series on herpes zoster following BNT162b2 COVID-19 vaccination in patients with autoimmune inflammatory rheumatic diseases. One of the six patients had herpes zoster ophthalmicus without corneal involvement. A 56-year-old female with a history of seropositive rheumatoid arthritis developed severe pain in the left eye and forehead four days after the first dose of BNT162b2 COVID-19 vaccine. The accompanying symptoms were hyperemic conjunctivitis of the left eye and a vesicular rash at the left forehead [12].

### 3.2. Cornea and Ocular Surface 

Six patients developed corneal and ocular surface manifestations after COVID-19 vaccination. The mean age of these patients was 68.5 (range 56–83) years old. The mean duration between COVID-19 vaccination and onset of ocular symptoms was 14.0 (range 7–21) days. The patients’ characteristics were reviewed in four articles.

- Corneal graft rejection after penetrating keratoplasty (PKP)

Ravichandran et al. reported a 62-year-old male with a history of PKP for a corneal scar in his right eye for two years. He had right eye congestion and blurred vision three weeks after the first dose of the ChAdO×1 COVID-19 vaccine. Khodadoust’s rejection line in the center of the endothelium with graft edema and anterior chamber (AC) reaction was noted. Corneal graft rejection of the right eye was diagnosed [13]. Wasser et al. reported two cases of corneal graft rejection after the first dose of BNT162b2 COVID-19 vaccine [14]. A 73-year-old male with a history of PKP in the left eye due to keratoconus and underwent regrafting due to late endothelial failure two years prior. He experienced left eye discomfort 13 days after vaccination. Left eye ciliary injection, corneal edema, Descemet folds, and keratic precipitates (KP) were noted, and corneal graft rejection was diagnosed. The other patient was a 56-year-old male with a history of bilateral keratoconus who underwent PKP 25 and seven years ago in the right and left eyes, respectively. Right eye regraft was performed due to late endothelial failure 10 months previously. He developed blurred vision in the right eye 14 days after vaccination. Right eye corneal edema, KPs, and AC cells were noted, and corneal graft rejection was diagnosed [14].

- Corneal graft rejection after Descemet membrane endothelial keratoplasty (DMEK)

Crnej et al. reported a 71-year-old male with a history of endothelial decompensation after phacoemulsification and who underwent DMEK of the right eye for five months [15]. He developed a decrease in vision seven days after the first dose of the BNT162b2 COVID-19 vaccine. A right eye conjunctival injection and corneal edema were noted. The central corneal thickness was 714 μm, and corneal endothelial graft rejection was diagnosed. Phylactou et al. reported two cases with a history of Fuchs endothelial corneal dystrophy who underwent DMEK with acute corneal endothelial graft rejection after administration of BNT162b2 COVID-19 vaccine [16]. A 66-year-old female who underwent DMEK of the right eye 21 days prior developed blurred vision seven days after the first dose of the vaccine. Right eye conjunctival injection, diffuse corneal edema, and fine KPs were noted, and corneal endothelial graft rejection of the right eye was diagnosed. Another 83-year-old female underwent DMEK six and three years prior in the right and left eyes, respectively. She had bilateral blurring of vision, pain, photophobia, and redness three weeks after the second dose of the vaccine. Bilateral circumcorneal injections, KPs, and AC inflammation were also noted. The central corneal thickness was 660 µm and 622 µm in the right and left eyes, respectively. A diagnosis of bilateral simultaneous acute endothelial graft rejection was made.

### 3.3. Retina 

In our literature review, there were seven patients who had retinal manifestations. The mean age of these patients was 24.7 (range 20–33) years old. The mean duration between COVID-19 vaccination and onset of retinal symptoms was 4.0 (range 1–15) days. The patients’ characteristics were reviewed in five articles.

- Acute Macular Neuroretinopathy (AMN)

Bøhler et al. reported a 27-year-old female who took oral contraceptives and developed an acute paracentral scotoma after receiving the first dose of the ChAdO×1 COVID-19 vaccine [18]. Fundoscopy of the left eye revealed a delicate teardrop-shaped macular lesion located nasally with respect to the fovea. Ocular coherence tomography (OCT) demonstrated slight hyperreflectivity of the outer nuclear and plexiform layers and disruption of the ellipsoid zone. Thus, AMN of the left eye was diagnosed. Gabkak et al. reported a 20-year-old female with a history of oral contraceptives and with bilateral flickering scotoma after administration of the ChAdO×1 COVID-19 vaccine [19]. Fundoscopy revealed subtle brightening around the fovea. OCT showed hyperreflective plaques and disruption of the ellipsoid junction. Therefore, bilateral AMN was diagnosed. Book et al. reported a previously healthy 21-year-old female with bilateral paracentral scotomas three days after receiving the first ChAdO×1 COVID-19 vaccine [20]. Infrared reflectance imaging showed bilateral circumscribed paracentral dark lesions that matched outer plexiform layer thickening and discontinuity of the ellipsoid band on OCT. A diagnosis of bilateral AMN was made. Mambretti et al. reported a 22-year-old female and a 28-year-old female who were on long-term oral contraceptives they developed acute onset of paracentral scotoma two days after receiving the ChAdO×1 COVID-19 vaccine. Multimodal retinal imaging was consistent with AMN in both cases [17].

- Central serous retinopathy

Fowler et al. reported a 33-year-old male who did not take any medications. He developed blurred vision and metamorphopsia in his right eye 69 h after receiving the BNT162b2 COVID-19 vaccine. OCT of the right eye showed serous macular detachment of the neurosensory retina. On fluorescein angiography (FA), a single point of leakage was noted following the ink-blot pattern. He was diagnosed with central serous retinopathy in the right eye [21].

- Retinal detachment

Subramony et al. reported a 22-year-old female with myopia but had no ocular trauma or any major past medical history. She developed vision loss in her right eye 15 days after the second dose of the mRNA-1273 COVID-19 vaccine. Fundoscopy revealed bilateral retinal detachment (macula-off in the right, macula-on on the left) despite having no vision loss in her left eye [22].

### 3.4. Uvea 

In our literature review, there were seven patients with uveal manifestations. The mean age of the patients was 35.2 (range 23–43) years old. The mean duration between COVID-19 vaccination and onset of uveitis symptoms was 15.2 (range 3–42) days. The patients’ characteristics were reviewed in six articles.

- Acute anterior uveitis

Renisi et al. reported that a 23-year-old male who had no significant medical history developed ocular symptoms after receiving the BNT162b2 vaccine. He developed unilateral periocular erythema, with involvement of the left eyelid 5 h after the first dose of the vaccine. The symptoms resolved 72 h after topical glucocorticoid administration. However, he developed blurred vision, red eye, and conjunctival hyperemia of the left eye 14 days after the second dose of the vaccine. Left eye posterior synechiae, AC cells, and KPs were noted. Funduscopic examination and autoimmune or infection lab screening did not reveal any alterations. A diagnosis of left eye acute anterior uveitis was made [23].

- Panuveitis

Mudie et al. reported a 43-year-old female who developed decreased vision three days after the second dose of the BNT162b2 COVID-19 vaccine. On examination, anterior chamber and vitreous inflammation were noted. However, she was also diagnosed with asymptomatic COVID-19 shortly after the onset of ocular symptoms [24].

- Multifocal Choroiditis

Goyal et al. reported a 34-year-old man had vision loss one week after receiving the second dose of the COVID-19 vaccine. On examination, serous detachment of the macula in the right eye and severe choroidal thickening noted on ultrasonography in both eyes were noted. A bilateral multifocal choroiditis was diagnosed [25].

- Acute zonal occult outer retinopathy (AZOOR)

In a case report on ocular inflammatory side effects after administration of COVID-19 vaccine, Maleki et al. reported a 33-year-old previously healthy female who developed progressive nasal field defect in her left eye and flashes in both eyes. Her symptoms occurred 10 days after receiving the second mRNA-1273 COVID-19 vaccine. Laboratory investigations demonstrated high erythrocyte sedimentation rate and C-reactive protein levels. On examination, there was no inflammation in either the anterior or vitreous cavities. OCT on both eyes showed a segmented and disrupted ellipsoid zone in the right eye and a very thin ellipsoid zone in the left eye. Multi-luminance-flicker electroretinogram revealed defective areas in the inferotemporal macula and temporal macula in the right and left eyes, respectively. A bilateral AZOOR was diagnosed [26].

- Reactivation of Vogt-Koyanagi-Harada (VKH) Disease

Papasavvas et al. reported that a 40-year-old female taking infliximab for VKH disease for six years had reactivation six weeks after the second dose of BNT162b2 COVID-19 vaccine. On examination, AC inflammation, mutton-fat KPs, retinal folds, subretinal fluid, and increased choroidal thickness were noted [27].

- Uveitis

Furer et al. conducted a multicenter observational study on the immunogenicity and safety of the BNT162b2 vaccine in adult patients with autoimmune inflammatory rheumatic diseases. The results showed that, among 686 cases, one case developed uveitis several weeks after the first dose of the vaccine and two cases developed uveitis after the second dose of the vaccine [28].

### 3.5. Nerve

In our literature review, there were three patients who developed neural manifestations. The mean age of the patients was 59.3 (range 40–79) years old. The mean duration between COVID-19 vaccination and onset of neural symptoms was six (range 2–14) days. Patients’ characteristics were reviewed in three articles.

- Optic neuritis

Helmchen et al. reported a 40-year-old female with a history of relapsing-remittent multiple sclerosis (MS) and developed bilateral blurring of vision that rapidly progressed to blindness two weeks after the first dose of the ChAdO×1 COVID-19 vaccine. Other symptoms include paraparesis that deteriorated to paraplegia, with absent tendon reflexes in the legs, incontinence, and a sensory deficit for all modalities below Th5 (thoracic spine). Brain magnetic resonance imaging (MRI) revealed numerous old white matter lesions compatible with MS and increased signal intensity in the chiasm and part of the adjacent optic nerves and tracts. Spinal MRI revealed myelitis at Th7–10, while aquaporin-4 (AQP4) was negative in serum and cerebrospinal fluid. Optic neuritis with AQP4-antibody negative neuromyelitis optica spectrum disorders-like syndrome was diagnosed [29].

- Arteritic anterior ischemic optic neuropathy (AAION)

Maleki et al. reported a 79-year-old previously healthy female who had a sudden bilateral loss of vision two days after the second dose of BNT162b2 COVID-19 vaccine. Laboratory examination revealed a very high erythrocyte sedimentation rate and C-reactive protein level. There was a 3+ afferent pupillary defect in the right eye. Fundoscopy revealed complete pallor of the optic nerve head in the right eye and segmental pallor in the left eye. OCT showed ganglion cell complex thinning, and the retinal nerve fiber layer appeared normal in both eyes. The visual field in the right eye showed severe generalized depression, and the left eye depicted a superior altitudinal defect. A right temporal artery biopsy was compatible with AAION, and bilateral AAION was diagnosed [26].

- Abducens nerve palsy

Reyes-Capo et al. reported that a 59-year-old previously healthy female had acute horizontal diplopia two days after receiving the BNT162b2 COVID-19 vaccine. On examination, right esotropia of 25 diopters in primary gaze, 30 diopters in the right gaze, 10 diopters in the left gaze, and a severe abduction limitation of the right eye were noted. MRI of the brain and orbit was unremarkable. The patient was diagnosed with right abducens nerve palsy [30].

### 3.6. Vascular Thrombosis 

In our literature review, there were eight patients with vascular thrombosis and related ocular manifestations. The mean age of the patients was 42.9 (range 18–60) years old. The mean duration between COVID-19 vaccination and onset of ocular symptoms was 8.1 (range 2–13) days. The patients’ characteristics were reviewed from eight articles.

- Superior ophthalmic vein (SOV) thrombosis

Panovska-Stavridis et al. reported a 29-year-old previously healthy female with blurring of vision of the left eye nine days after receiving the first dose of the ChAdO×1 COVID-19 vaccine. The accompanying symptoms were severe headache, left eye swelling with proptosis, limited ocular motility, and diplopia at the vertical gaze. Laboratory investigations revealed thrombocytopenia and high D-dimer levels. IgG enzyme-linked immunosorbent assay (ELISA) for platelet factor 4 (PF4)-heparin complex antibodies was positive. MRI demonstrated left SOV thrombosis with widening SOV and filling defects [31]. On the other hand, Bayas et al. reported a 55-year-old previously healthy female who developed conjunctival congestion, retro-orbital pain, and diplopia 10 days after receiving the first dose of ChAdO×1 COVID-19 vaccine. Laboratory investigations revealed thrombocytopenia. IgG-ELISA for PF4-heparin complex antibodies was negative. MRI showed SOV thrombosis with no contrast filling [32].

- Cerebral venous sinus thrombosis (CVST)

Castelli et al. reported that a 50-year-old previously healthy male developed slight visual impairment 11 days after receiving the first dose of the ChAdO×1 COVID-19 vaccine. He also had severe headache, slight deviation of the right buccal rim, loss of strength in the right lower limb, and unstable walking. Laboratory investigations revealed decreased platelet count and lack of fibrinogen. Computed tomography (CT) angiography showed multiple parenchymal hemorrhage and thrombosis of the left transverse and sigmoid sinuses [33]. Wolf et al. reported a 46-year-old previously healthy female who developed headache, hemianopia to the right, and aphasia 13 days after receiving the ChAdO×1 COVID-19 vaccine. Laboratory examination revealed thrombocytopenia. IgG-ELISA for the PF4-heparin complex antibody was positive. MRI showed thrombotic occlusion of the superior sagittal sinus and the left-hand transverse sinus and sigmoid sinus. Left occipital intracerebral hemorrhage was also noted [34]. Suresh et al. reported a 27-year-old male who developed headache associated with eye floaters and vomiting 48 h after receiving the first dose of the ChADO×1 COVID-19 vaccine. Laboratory investigations revealed elevated D-dimer levels, low platelet counts, and fibrinogen levels. IgG-ELISA for the PF4-heparin complex antibody was positive, while CT venogram showed CVST. However, the headache worsened, and new homonymous hemianopia occurred the following day. Repeat CT showed right parietal lobe hemorrhage [35]. Dias et al. reported a 47-year-old female with a history of iron-deficiency anemia due to adenomyosis and oral contraceptives. She developed persistent headache, nausea, and photophobia six days after the first dose of the BNT162b2 COVID-19 vaccine. Three days after the consult, she experienced sudden left hemiparesis. Papilledema, left visual extinction, and right gaze deviation were noted. Laboratory investigations revealed a normal platelet count. Brain MRI with venography revealed CVST with right frontal subarachnoid hemorrhage and a cortical venous infarct. IgG-ELISA for PF4-heparin complex antibodies, measured two months after the event, were negative [36]. See et al. conducted a case series of six patients with CVST with thrombocytopenia after receiving the Ad26.COV2.S vaccine. A female patient experienced headache and visual changes six days after vaccination. After 10 days, she developed ecchymoses, periorbital and lower extremity petechiae, and intermittent shortness of breath. Seventeen days after vaccination, she suddenly lost consciousness, and thrombocytopenia was noted. Brain magnetic resonance imaging (MRI) revealed CVST and right internal jugular vein thrombosis [37].

- Thrombocytopenia with acute ischemic stroke and bleeding

Blauenfeldt et al. reported that a 60-year-old female with a history of Hashimoto thyroiditis and hypertension developed abdominal pain seven days after receiving the first dose of the ChADO×1 COVID-19 vaccine. Laboratory investigations showed thrombocytopenia, raised D-dimer, and she was positive for PF4 antibodies. Abdominal computed tomography (CT) revealed bilateral adrenal hemorrhages and a subcapsular renal hematoma. The following day, she developed left-sided weakness and eye deviation to the right. MRI revealed a complete infarction in the entire area supplied by the right middle cerebral artery. The patient died on the sixth day of hospitalization [38].

### 3.7. Other Reports with Only Ocular Symptoms Mentioned

In our literature review, there were 12 patients with ocular manifestations. The duration between COVID-19 vaccination and onset of ocular symptoms ranged from three days to six weeks. The patients’ characteristics were reviewed from two articles.

Kadali et al. conducted a randomized, cross-sectional study to investigate the side effects of the BNT162b2 COVID-19 vaccine in 803 healthcare workers. Among them, four (0.5%) participants had blurring of vision and seven (0.87%) participants had eye pain within six weeks after receiving the vaccine [40]. Santovito et al. reported that a middle-aged, previously healthy male developed a sudden onset of darkening of the visual field three days after receiving the second dose of BNT162b2 COVID-19 vaccine. He also developed light confusion, asthenia, and profound nausea [39].

## 4. Discussion

Our review identified multiple ocular manifestations following COVID-19 vaccination. The COVID-19 vaccines included in these studies are mRNA vaccines and adenoviral vector vaccines, both of which do not contain the spike protein. Instead, these contain genetic information about the spike protein for its biosynthesis in body cells [41].

### 4.1. mRNA Vaccines

The mRNA vaccines, including BNT162b2 and mRNA-1273, contain codon-optimized sequences for spike protein synthesis and use the authentic signal sequence for its biosynthesis [41]. The mRNA is delivered in lipid nanoparticles and induce the production of proinflammatory cytokines, such as interferon gamma (IFNγ), antigen-specific CD4+ and CD8+ T cell responses, and anti-spike neutralizing antibody. IFNγ-producing CD4+ T helper 1 (Th1) and virus antigen-specific T cells may induce corneal allograft rejection [14,15,16]. mRNA vaccines can also stimulate innate immunity through toll-like receptors, which are related to reactivation and maintenance of herpesviruses in the host [12]. The mRNA vaccine can increase free extracellular mRNA, which may increase the permeability of endothelial cells and cause choriocapillaris leakage. Moreover, mRNA vaccines may trigger endogenous glucocorticoid release to increase serum cortisol levels. Lipid nanoparticles consisting of polyethylene glycol, which may induce activation of the complement pathway, could lead to choroidal vessel thickening and neovascularization. All these factors may contribute to CSCR [21]. 

The mRNA vaccine can also induce antigen-specific cell-and antibody-mediated hypersensitivity reactions and molecular mimicry due to the close resemblance between the vaccine peptide fragments and uveal self-peptides [23,42]. These immune reactions play an important role in the formation of uveitis. Similarly, antibodies against spike proteins and activated Th1 cells can cross-react with proteins and antigens in the outer retinal layers, retinal pigment epithelial cells, and large arteries, which may induce AZOOR and AAION [26]. Other possible effects induced by mRNA vaccines include microangiopathy, localized vasculitis, and demyelination, which cause the presence of eyelid purpuric and ecchymotic lesions and abducens nerve palsy [11,30].

### 4.2. Adenoviral Vector Vaccines

Adenoviral vector vaccines, including ChAdO×1—chimpanzee adenovirus Y25 and Ad26.COV2.S—human adenovirus 26, use replication-incompetent adenovirus as a vector to deliver the DNA signal sequence of spike protein [43,44]. However, evidence has shown that during transcription, alternative splice events might cause the formation of C-terminally truncated, which may lead to formation of soluble spike protein. These soluble spike variants may induce thrombotic events via an antibody-mediated mechanism when binding to ACE2-expressing endothelial cells in blood vessels [41,45]. Other possible causes of thrombosis include spike protein interactions with different C-type lectin receptors, heparan sulfate proteoglycans, and the CD147 receptor. Lastly, adenovirus vectors may interact with the CD46 receptor or platelet factor 4 antibodies to cause thrombosis [1].

Thrombocytopenia syndrome (TTS), a rare syndrome that involves acute vessel thrombosis, has been reported in numerous studies in patients receiving COVID-19 adenoviral vector vaccines. MacIntyre et al. reported that the ChAdO×1 COVID-19 vaccine is associated with TTS in three of 100,000 vaccinations [46]. Moreover, 15 cases of TTS were reported in the United States after administration of 7.98 million doses of Ad26.COV2.S COVID-19 vaccination during 2 March–21 April 2021 [47]. Although the mechanism of TTS is not clear, the clinical course and laboratory test results of TTS are similar to autoimmune heparin-induced thrombocytopenia which is triggered by the formation of PF4 antibodies [37,47]. The TTS occurred mostly in middle aged women 5–24 days after vaccination. The platelet count ranged from 7000 to 113,000 platelets per cubic millimeter, and most cases were positive for PF4-heparin antibodies [48]. The TTS can cause thrombus formation in arteries or veins, which might lead to CVST and SOV thrombosis. By 4 April 2021, 169 cases of CVST were reported among 34 million people vaccinated in the European Economic Area and United Kingdom with the ChAdO×1 COVID-19 vaccine [48]. According to our reviewed cases, the disease can present as headache with blurred vision, limited ocular motility, diplopia, strabismus, hemianopia, or papilledema [33,34,35,36,37,38].

Adenoviral vector vaccines can cause ocular disease by inducing an immunologic response to the spike antigen or to components of the chimpanzee or human adenovirus. Retinal pathologies may be caused by molecular mimicry and self-antigens. Furthermore, the expressed spike antigen in the circulation can directly cause disturbance in vascular endothelial cells or induce a proinflammatory and procoagulant response, which leads to AMN. However, four cases in our review had a history of long-term oral contraceptives use, which are known as possible risk factors for AMN [18,49]. Finally, the dysimmunological process may be triggered by the adenoviral vector-targeted B cell immune response. The immune reaction can induce the reactivation of optic neuritis with longitudinal extensive transverse myelitis in longstanding stable MS [29]. The relationship between adenoviral vector vaccines and all of the ocular symptoms mentioned should be further investigated. We agreed with Ng et al. that the pathogenesis of the ocular symptoms after COVID-19 vaccination may be the immune response toward the vaccine [50]. Thus, other studies are needed to prove a causal link between the observed event and the vaccine, which may contribute to future vaccine development.

## 5. Conclusions

The reviewed studies raised our concern for the ocular manifestations after COVID-19 vaccination. However, the overall benefits of the COVID-19 vaccine in preventing COVID-19 are well established. The incidence rate of ocular manifestations after receiving the vaccine is considerably lower than the prevalence rate of ocular symptoms in COVID-19 patients [51]. In conclusion, although ocular manifestations may develop after receiving the COVID-19 vaccine, people are still encouraged to get vaccinated since the benefits outweigh the risks. 

## Figures and Tables

**Table 1:** Review of literature of ocular manifestations after COVID-19 vaccine.

Study	Type	Rating Score *	Age (Years)	Sex	Vaccine	Dose	Duration between Vaccine and Ocular Symptoms (Days)	Diagnosis
Al Khames Aga et al. [10]	Retrospective cohort	3	N.A.	N.A.	BNT162B2	N.A.	1	Eyelid swelling and allergy
Mazzatenta et al. [11]	Letter to editor	5	44 63 67	F M F	BNT162B2 BNT162B2 BNT162B2	2nd 2nd 1st	21–25 21 10	Eyelid purpuric lesions Eyelid purpuric lesions Eyelid ecchymotic lesions
Furer et al. [12]	Case series	4	56	F	BNT162B2	1st	4	Herpes zoster ophthalmicus
Ravichandran et al. [13]	Case report	5	62	M	ChAdOx1	1st	21	Corneal graft rejection
Wasser et al. [14]	Case report	5	73 56	M M	BNT162B2 BNT162B2	1st 1st	14 14	Corneal graft rejection Corneal graft rejection
Crnej et al. [15]	Letter to editor	5	71	M	BNT162B2	1st	7	Corneal graft rejection
Phylactou et al. [16]	Case report	5	66 83	F F	BNT162B2 BNT162B2	1st 2nd	7 21	Corneal graft rejection Corneal graft rejection
Mambretti et al. [17]	Case report	5	22 28	F F	ChAdOx1 ChAdOx1	N.A. N.A.	2 2	Acute macular neuroretinopathy Acute macular neuroretinopathy
Bøhler et al. [18]	Letter to editor	5	27	F	ChAdOx1	1st	2	Acute macular neuroretinopathy
Gabka et al. [19]	Case report	5	20	F	ChAdOx1	N.A.	1	Acute macular neuroretinopathy
Book et al. [20]	Images	5	21	F	ChAdOx1	1st	3	Acute macular neuroretinopathy
Fowler et al. [21]	Case report	5	33	M	BNT162B2	N.A.	3	Central serous retinopathy
Subramony et al. [22]	Case report	5	22	F	mRNA-1273	2nd	15	Retinal detachment
Renisi et al. [23]	Case report	5	23	M	BNT162B2	2nd	14	Acute anterior uveitis
Mudie et al. [24]	Case report	5	43	F	BNT162B2	2nd	3	Panuveitis
Goyal et al. [25]	Case report	5	34	M	ChAdOx1	2nd	7	Multifocal choroiditis
Maleki et al. [26]	Case report	5	33 79	F F	mRNA-1273 BNT162B2	2nd 2nd	10 2	Acute zonal occult outer retinopathy Arteritic anterior ischemic optic neuropathy
Papasavvas et al. [27]	Case report	5	43	F	BNT162B2	2nd	42	Reactivation of Vogt-Koyanagi-Harada disease
Furer et al. [28]	Case-control study	3	N.A.	N.A.	BNT162B2	1st/2nd	N.A.	Uveitis
Helmchen et al. [29]	Letter to editor	5	40	F	ChAdOx1	1st	14	Optic/chiasm neuritis with longitudinal extensive transverse myelitis
Reyes-Capo et al. [30]	Case report	5	59	F	BNT162B2	N.A.	2	Abducens nerve palsy
Panovska-Stavridis et al. [31]	Letter to editor	5	29	F	ChAdOx1	1st	9	Superior ophthalmic vein thrombosis
Bayas et al. [32]	Images	5	55	F	ChAdOx1	1st	10	Superior ophthalmic vein thrombosis
Castelli et al. [33]	Letter to editor	5	50	M	ChAdOx1	1st	11	Cerebral venous sinus thrombosis
Wolf et al. [34]	Case series	4	46	F	ChAdOx1	1st	13	Cerebral venous sinus thrombosis
Suresh et al. [35]	Case report	5	27	M	ChAdOx1	1st	2	Cerebral venous sinus thrombosis
Dias et al. [36]	Case report	5	47	F	BNT162B2	1st	6	Cerebral venous sinus thrombosis
See et al. [37]	Case series	4	18–39	N.A.	Ad26.COV2.S	1st	6	Cerebral venous sinus thrombosis
Blauenfeldt et al. [38]	Case report	5	60	F	ChAdOx1	1st	8	Acute ischemic stroke and bleeding
Santovito et al. [39]	Letter to editor	5	middle-aged	M	BNT162B2	2nd	3	N.A.
Kadali et al. [40]	Cross sectional study	4	N.A.	N.A.	BNT162B2	N.A.	1–42	N.A.

* Rating score of the studies was ranked according to Quality Rating Scheme for Studies and Other Evidence [8] and Oxford Centre for Evidence-based Medicine for ratings of individual studies [9]. Abbreviations: F female, M male, N.A. not mentioned in the article.
